# Supplementary material for: Combining N-mixture and occupancy analysis offers a more complete picture of carnivore habitat use in Northeastern Türkiye
Source: PLoS One. 2025 May 14;20(5):e0320768. doi: 10.1371/journal.pone.0320768 (PMC12077724; doi:10.1371/journal.pone.0320768)
Supplement: Attachment — Submitted filename: PONE-D-23-35903_R1_review.pdf [file pone.0320768.s007.pdf]

Review for:

Combining N-mixture and Occupancy analysis offers a more complete picture of carnivore habitat use in northeastern Türkiye

NOTE: This review is written in markdown format. I've also included a pdf if that is easier for the authors to read.

In this manuscript the authors assembled an incredibly impressive dataset to assess how inference may vary between n-mixture models and occupancy models. In addition, they also assessed how increasing the sampling window for the repeat surveys may affect results across these two models. Overall, I think the writing in this manuscript is good. As this is a revision, I looked through the responses and would say that the authors did a fair job responding to them. However, some aspects that the reviewers of the previous draft brought up still exist in this draft (see below). As these issues are related to presentation, they should be able to be addressed, it is just that some aspects of the methods especially need to be made more clear.

With respect to showcasing differences between occupancy and n-mixture models, I think the authors did fine. The species-specific figures that show the results across model types are interesting though a little hard too unpack.

I do, however, have an issue with the survey length part of the analysis. As I showcase this throughout the review in multiple spots I will be brief right here, but the increased abundance from the n-mixture models as survey length increases is the result of violating the assumption that individuals are not double counted. This is not explained at all in the manuscript, and as a result, I would fear that people reading this paper may misinterpret the findings, which could have an effect on how others conduct such analyses.

To be more specific about what I brought up above:

1. There are still some issues identified by the previous reviewers that exist in this draft. I, for example, had a lot of trouble determining what covariates were used within each model given Table 1 and the 'covariate selection' section. That specific issue is not insurmountable, but readers should be able to clearly and easily understand the model fitting procedure, which includes which covariates were used where.

2. The paper lacks any justification for why varying detection windows are used, and why we would want to compare these across models. I think this comparison could be useful, but the paper needs to set the foundation for why this matters, and at the moment I feel like this responsibility is being left with the reader (which is not ideal).

3. This is related to the previous comment, but I have moved this to a different section there is a bit to unpack here. Basically, I'm left wondering why a shift in detection probability, which comes with a decrease in the number of secondary surveys that could occur, results in an increase in counts from the N-mixture model. I'm assuming that the binning process here results in greater counts in the observed data, which may be causing this? This, to me, indicates a rather glaring violation of an assumption for this class of model, which is that counts are unique (i.e., an individual is not double counted).

To demonstrate that this is an issue with the data summarizing process, I ran a simulation. Specifically, I simulated a simple data set and fitted a single season n-mixture model coded up in `nimble`. First, I simulated data for 150 sites and 8 replicates per site. From there, I converted those 8 replicates into 4 by summing replicate 1 & 2, 3&4, etc. etc. This is similar in spirit to your binning process, as the counts would be independent under your count assumptions (photos one minute apart, which would certainly happen over multiple replicate camera trap days). Based on the results from the simulation study, it seems to me that the shift in expected counts is 100% related to the binning process itself (i.e., the observed counts are not independent because some individuals have been counted twice, but the model assumes independence at a 1 minute interval between camera trap images of the same species). As such, I feel as if the binning process used here is creating the issues with the model fitting as the counts supplied to the model are increasingly violating a model assumption (i.e., 10-day duration window is worse than durations less than that). See Link et al. (2018), which goes into assumption violations from this class of model. Moreover, others have used much larger binning methods (this paper used 1 minute difference between subsequent images of the same species) and that may limit this specific issue (Koetke et al. 2024). For example, you may want to consider using lorelograms to figure out the appropriate window to reduce temporal correlation in the photo data, which may lead to less biased estiamtes (Iannarilli et al. 2019).

Iannarilli, F., Arnold, T. W., Erb, J., & Fieberg, J. R. (2019). Using lorelograms to measure and model correlation in binary data: Applications to ecological studies. Methods in Ecology and Evolution, 10(12), 2153-2162.

Link, W. A., Schofield, M. R., Barker, R. J., & Sauer, J. R. (2018). On the robustness of N-mixture models. Ecology, 99(7), 1547-1551.

Koetke, L. J., Hodder, D. P., & Johnson, C. J. (2024). Using camera traps and N-mixture models to estimate population abundance: Model selection really matters. Methods in Ecology and Evolution, 15(5), 900-915.

Code to recreate the simulations I ran

```
library(nimble)
library(MCMCvis)

set.seed(154)

# number of sites
n <- 150
```

```r
# number of replicates
j <- 8

# intercept only model
lambda <- log(10)

# latent count
y <- rpois(
  n,
  exp(lambda)
)

# low detection prob at j = 8 replicates
rho <- 0.1

y_obs <- matrix(
  NA,
  ncol = j,
  nrow = n
)

for(i in 1:j){
  y_obs[,i] <- rbinom(
    n,
    y,
    rho
  )
}


my_model <- nimble::nimbleCode({
  lambda ~ dnorm(0, 2)
  rho ~ dlogis(0,1)
  for(site in 1:nsite){
    log(log_lambda[site]) <- lambda
    y_latent[site] ~ dpois(log_lambda[site])
    for(rep in 1:nrepeat){
      logit(det_prob[site,rep]) <- rho
      y_observed[site,rep] ~ dbin(
        det_prob[site,rep],
        y_latent[site]
      )
    }
  }
})

data_list <- list(
  y_observed = y_obs
)
constant_list <- list(
  nsite = n,
  nrepeat = j
)

# fit with the j = 8 repeats
model_j8 <- nimble::nimbleMCMC(
  code = my_model,
  data = data_list,
  constants = constant_list,
  monitor = c("y_latent", "rho", "lambda"),
  niter = 40000,
  nburnin = 10000,
  nchains = 3
)

j8_summary <- MCMCvis::MCMCsummary(
  model_j8,
  round = 2
)
# see if they converged (yes)
range(j8_summary[,6])

tail(j8_summary)
```

```r
y_obs_j4 <- matrix(
  NA,
  ncol = 4,
  nrow = n
)
col_vec <- matrix(1:8, ncol = 4, nrow = 2)

for(i in 1:4){
  y_obs_j4[,i] <- rowSums(
    y_obs[,col_vec[,i]]
  )
}


data_list2 <- list(
  y_observed = y_obs_j4
)
constant_list2 <- list(
  nsite = n,
  nrepeat = ncol(y_obs_j4)
)

# fit with the j = 8 repeats
model_j4 <- nimble::nimbleMCMC(
  code = my_model,
  data = data_list2,
  constants = constant_list2,
  monitor = c("y_latent", "rho", "lambda"),
  niter = 40000,
  nburnin = 10000,
  nchains = 3
)

j4_summary <- MCMCvis::MCMCsummary(
  model_j4,
  round = 2
)
# we would expect rho to be different between models,
#  but the other variables should not especially change.

# plot estimates
to_plot <- data.frame(
  j8 = j8_summary[-c(1:2),1],
  j4 = j4_summary[-c(1:2),1]
)

# you end up with higher abundance with the four replicate
#  binning process (all points under line).
plot(
  to_plot$j8 ~ to_plot$j4,
  ylim = c(5,20),
  xlim = c(5,20),
  xlab = "Four replicates",
  ylab = "Eight replicates"
)
abline(a = 0, b = 1)
```

In the rest of this document I go through each section, providing top-level and line-by-line comments as needed. I hope the authors find them useful. If you have any questions, I can be reached at mfidino@lpzoo.org.

- Mason Fidino

Abstract

Increasing the detection window does not actually increase the detection probability, it is simply that because the window has gotten bigger the cumulative probability has changed. Take, for example, a daily detection probability of 0.05, which is quite low but relatively common to see when analyzing camera trap data in an occupancy analysis. If I modified my detection window from a day to a week, the detection probability goes up, but only because I am now quantifying 'the probability of detecting the species at least once given a week of sampling' rather than 'the probability of detecting the species at least once given a day of sampling.' In fact, you can use general probability theory to see what a species weekly detection probability is given their daily detection probability. With a 0.05 daily detection probability, the probability you detect a species given a week (i.e., seven days) of sampling is 1 - (1 - 0.05)^7, which is just about 0.30. The detection probability across varying detection windows are therefore equivalent and so while you are correct that 'increasing the detection window generally increases the detection rate,' it has

nothing to do with the species and everything to do with interpretation. However, increasing the detection window can help deal with boundary estimation issues. When daily detection probabilities are exceedingly low, which can happen, it is difficult to quantify variation in this probability (i.e., estimate slope terms) because the probability is so near zero. I bring this up here simply because I would not want someone to read this and interpret the statement being made incorrectly. The detection probability HAS to go up when you increase the detection window.

Introduction

Top-level thoughts

1. The distinction that the introduction makes between occupancy models and n-mixture models, especially as applied to camera trap data, is perhaps not as distinct at the paper tries to make it. The observational level of the detection model can still be interpreted as intensity of use (e.g., a species is detected at one site on two surveys while at another site of 10 surveys). This can be especially helpful when a species occupies close to the entire landscape (e.g., Gallo et al. 2018).

> Gallo, T., Lehrer, E. W., Fidino, M., Kilgour, R. J., Wolff, P. J., and Magle, S. B. (2018). Need for multiscale planning for conservation of urban bats. Conservation Biology, 32(3):638–647. PDF

2. There really is not much justification why the repeat sampling window duration was modified. There has been some very recent research that has looked into this as a way for accounting for temporal autocorrelation in a given detection history (Goldstein et al. 2024).

> Goldstein, B. R., Jensen, A. J., Kays, R., Cove, M. V., McShea, W. J., Rooney, B., ... & Pacifici, K. (2024). Guidelines for estimating occupancy from autocorrelated camera trap detections. Methods in Ecology and Evolution.

3. The hypothesis is one that would be pretty hard to disprove, which is something the reviewer in the last version of this manuscript pointed out. I think that predictions were provided that help flush this hypothesis out more, but the hypothesis on it's own still needs improvement. Species should be expected to vary in habitat use, modeling different types of data with different link functions should result in different results. If you want to use a hypothesis here, then I would instead try to focus on the comparison. How do you think occupancy and n-mixture models compare? Do you think one will outperform another? Will that relationship vary based on the ecology of a species? Data density? It's 100% okay for a hypothesis to be wrong, but in it's current form this specific hypothesis is not all that falsifiable given that the null would here is 'species use habitat the same way and these models work the same' which we don't need this study for to pretty reliably reject.

In fact, do you really need a hypothesis here? You've already stated two major questions (line 84 - 85), why not just focus on answering those?

Line by line comments

line 62: repeat-sampling observational data is perhaps not the most appropriate phrase to capture what is meant here. Maybe instead this sentence could be something like:

> For example, occupancy (10) and N-mixture models (11-13) are two commonly used statistical frameworks that can quantify habitat use while also accounting for the vagaries of sampling (e.g., imperfect detection).

or some derivative of this. No need to get into the camera trap side of things yet in my opinion, plenty of time for that later.

Line 73 - 77: If you want to combine the ecological description of the problem with a statistical description, you could indicate that when species are rare or common occupancy models have boundary estimation issues (i.e., trying to estimate probabilities close to 0 or 1). Clipp et al. (2021) talk about this problem in the case of multi-species models, but the idea generally applies.

> Clipp, H. L., Evans, A. L., Kessinger, B. E., Kellner, K., & Rota, C. T. (2021). A penalized likelihood for multispecies occupancy models improves predictions of species interactions. Ecology, 102(12), e03520.

Line 79 - 80: Here are two papers that have applied both types of models to the same data set.

> Ward, R. J., Griffiths, R. A., Wilkinson, J. W., & Cornish, N. (2017). Optimising monitoring efforts for secretive snakes: a comparison of occupancy and N-mixture models for assessment of population status. Scientific Reports, 7(1), 18074.
>
> Rosenbaum, D., Saenz, D., Montaña, C. G., Zhang, Y., & Schalk, C. M. (2023). Detection, occupancy, and abundance of the alligator snapping turtle in Texas. The Journal of Wildlife Management, 87(5), e22409.

Methods

Top-level thoughts

1. In the review response the authors posit that 'The Bayesian framework does not provide for a good way to do model selection.' Given the large number of papers that use information theoretic approaches to compare Bayesian models and ecology papers that have assessed how Bayesian model selection applies to latent state models, this response from the authors is not that accurate. Certainly, Bayesian model selection is a bit more tricky as you often have

to code up the likelihood yourself, but it is still something that can be done. I'd encourage the authors to check out Hooten and Hobbs (2015), which has a lot of great information on Bayesian model selection, as well as Broms et al. (2016). I've used various kinds of Bayesian model selection in my own research with occupancy models, and data / code for those papers are publicly available (I provide a link below with the most relevant example).

Is model selection necessary here? I'm not sure. Right now, based on the methods, I am left uncertain what covariates were used in the latent state part of each model. I think the reason for this it is a bit difficult to figure that out as readers must refer to table 1 and then remove slope and ruggedness, and perhaps others as they are reported in the covariate selection part oft he methods. This part of the model should be crystal clear. Easiest fix would be to add one more column to table 1 related to whether or not a given covariate was used (and please change the title to table 1 as not all of these covariates are used it seems).

```
Hooten, M. B., & Hobbs, N. T. (2015). A guide to Bayesian model selection for ecologists. Ecological monographs, 85(1), 3-28.

Broms, K. M., Hooten, M. B., & Fitzpatrick, R. M. (2016). Model selection and assessment for multi-species occupancy models. Ecology, 97(7), 1759-1770.
```

Bayesian Model selection stuff

Calculating the Conditional Predictive Ordinate (CPO) for single-season occupancy models (Fidino et al. 2020).

https://github.com/mfidino/uwin_ms_2/blob/8164972985a056f774552f182cbd54ef52376659/jags_scripts/city_mean_occupancy.R#L58-L67

Citation

```
Fidino, M., Gallo, T., Lehrer, E. W., Murray, M. H., Kay, C., Sander, H. A., MacDougall, B., Salsbury, C. M., Ryan, T. J., Angstmann, J. L., Belaire, J. A., Dugelby, B., Schell, C., Stankowich, T., Amaya, M., Drake, D., Hursh, S. H., Ahlers, A. A., Williamson, J., Hartley, L. M., Zellmer, A. J., Simon, K., and Magle, S. B. (2021). Landscape-scale differences among cities alter common species' responses to urbanization. Ecological Applications, 31(2):e02253.
```

2. The mathematical notation here could be improved a bit. A lot of this is not using italics or bold when needed, misuse of capitalization, using * to represent multiplication, and incorrect matrix algebra. Methe et al. (2019) is a wonderful resource for this. I've pointed out issues as I've noticed them in the line by line comments here, but I encourage the authors to give the math another close look just in case I missed anything.

```
Edwards, A. M., & Auger-Méthé, M. (2019). Some guidance on using mathematical notation in ecology. Methods in Ecology and Evolution, 10(1), 92-99.
```

3. Occupancy models inherently account for number of sample days in their likelihood and therefore it is redundant to include number of active days as a covariate in the analysis. If one site has 4 days of sampling and another has 40, the detection-level of the model would use 4 Bernoulli trials for the former and 40 for the latter (i.e., you have more coin flips in which to detect the species with 40 sampling days, you don't need to include the 40 as a covariate). For example, why would sampling for 40 days increase your detection probability on the first day? Your model indicates that it could, and I cannot think of a reason why that would increase the daily detection probability. Again, assuming a 0.05 daily detection probability, the probability of detecting a species at least once over 4 days of sampling is 1 - (1-0.05)^4 = 0.185 while for 40 days it is 1 - (1-0.05)^40 = 0.87. The increase in detection probability should be from the increased number of trails, but the number of trials that occurs has no bearing on the survey-specific detection probabilities. As a simple example, flipping a coin 5 vs 10 times does not increase the probability of getting a heads on each trial. With 10 flips you have a higher probability of getting heads at least once, but again the number of flips has no bearing on the individual probabilities.
4. I would still encourage using some covariates that may capture variation in local abundance in the detection part of the occupancy model. Local abundance does, after all, influence detection probability (McCarthy et al. 2013).

```
McCarthy, M. A., J. L. Moore, W. K. Morris, K. M. Parris, G. E. Garrard, P. A. Vesk, L. Rumpff, K. M. Giljohann, J. S. Camac, S. S. Bau, T. Friend, B. Harrison, and B. Yue. 2013. The influence of abundance on detectability. Oikos 122:717–726
```

5. I think it would help to remind the reader that sites are not sampled consistently throughout the entire length of the study, which is why you did not account for temporal autocorrelation in local site use intensity or occurrence in your models. Right now, I could see people reading this and thinking that this is the standard way to model these relationships, and in fact it is only applicable to this specific use case. It does make me wonder though how often sites are repeatedly sampled across successive years.

6. To build off the last comment. Since there are 13 years of data, it is strange to me that neither model has a subscript related to time. This makes things a bit confusing, as what happens if a site is sampled more than once over this 13 year period? Is it treated as a new site? How does this affect the definition of your site i subscript? Some additional clarity here would be helpful as I am a bit puzzled with how your data connects to these models.

7. What are your priors for intercept and slope terms in your multi-species model? With 3 species, having species-level random intercepts and slopes is a little difficult, though you could simply just give them all independent priors (i.e., not estimate a community level average).

NOTE: line 265 - 269 details this. While you can fit a standard multi-species model here, I would be very careful with trying to make inference on any community level averages or the standard deviation term (see Gomes 2022).

```
Gomes, D. G. (2022). Should I use fixed effects or random effects when I have fewer than five levels of a grouping factor
in a mixed-effects model?. PeerJ, 10, e12794.
```

Line by line comments

Line 158: Why exclude data when there were fewer than 7 active days? Those sites still provide information and the detection probability there is partially informed by sites that share similar habitat characteristics (so long as they are included in the detection part of the model). I've also subset data like this in the past, but have since realized that this is not necessary and is a bit arbitrary. Why 7 days and not 8? Why not 14 days? Excluding a bear rubbing tree makes sense though.

Line 170: In the review response there was a lot of focus on using canopy cover rather than distance to forest edge. Table 1 has the latter but not the former.

Line 191 - 192: May want to be explicit about what you mean by detection rate here (e.g., was it the number of secondary surveys detected / total number of secondary surveys or number of independent photos / total number of days sampled). The former is bound between 0 and 1 whereas the latter would not be, so some specificity here would be nice.

Line 221: N_{i,k} should be lowercase and italic (here and throughout).

Line 223: \lambda_{i,k} should be italic (here and throughout).

Line 221: define your subscripts and their range. For *i* in 1,...,*I* sites and *k* in 1,...,*K* species...

Line 223: k subscript should be italic (...species *k* detected...)

Line 225: italics for \alpha terms. Furthermore, you can just simplify this to:

```
$$
\text{log}(\lambda_{i,k}) =  \bf{a}_k \bf{x}_i
$$
```

where **a_k** is a vector of species-specific parameters and **x_i** is a conformable vector of site-level covariates where the first element is a 1 to account for the model intercept.

Right now it looks like you are multiplying a scalar (a_covs,k) by a matrix (capital X), which is confusing. Likewise, * does not represent multiplication (though there are plenty of ecology papers that treat it as such).

Line 230: uppercase Y should be y (and italics).

Line 232: change P to \rho (looks like a little p) and make it italic. Uppercase P is often reserved for the probability of some occurrence / event (i.e., P(event)).

Line 233: italics to subscripts.

Line 235: Logit is lowercase. \beta and \epsilon should be italicized.

Line 239: Missing Normal before your distribution.

Line 240 - 241: This is false (see above).

Line 246: Z should be lowercase and in italics.

Line 248: \psi should be italicized.

Line 250: see comments above, they apply here as well. Also, you've used these terms before (\alpha and x). They are not shared among models, so either remind the reader about how while the notation is similar the parameters are not shared or just use different symbols / letters. I prefer the latter so that it cannot be misinterpreted.

Line 258: Y should be lowercase and italics. And again, y means different things across models so this is inviting confusion. Finally, see comment about asterisk above (in fact, just drop it, p_{ijk}z{ik} already represents the product).

Line 275: how long was the burn-in? Reading that sentence makes me think the burn-in was 2 steps.

Results

Top-level thoughts

1. Again, detection probability must increase as the sampling window increases (i.e., the sampling duration).
2. I'm uncertain why inference is being made across all different sampling duration models. This is not really explained that well in the paper, and it leads to a lot of uncertainty in the results about how to unpack all these relationships. What do you do, for example, when the 10-day model finds 'significant effects' whereas the others do not? Furthermore, does average mean effect, as reported in lines 413 - 422 for example, represent the average across sampling duration models? This is unclear. If this is the case, I would caution against doing so.
3. It's a bit easier to have commas separate the values in your credible interval, as the double negatives get a little weird. Take for example, line 438

```
...effect: -0.34, range: -0.43 - -0.27; mean SD: 0.21, ...
```

I would separate all these values with semi-colons, and then use commas within a element in the list as needed.

```
...effect: -0.34; range: -0.43, -0.27; mean SD: 0.21; ...
```

4. The species level effects have an incredibly long list of values reported for them, and it makes it very difficult to read and compare. I'm not 100% sure what the answer is here, but I had a very difficult time trying to connect the results listed there back to the species themselves. Perhaps if there was some reasoning as to what all these values need to be reported, that would help, otherwise it may be simpler to move those to a supplementary table and focus in on the key results instead.

Line by line comments

Line 384 - 385: If you are going to provide evidence of an effect this way, add it to the methods as well instead of right here.

Line 490 - 492: count data is much more rich in terms of information than binary data, so that is not too surprising (but good to see here this occur in the results).

Discussion

Top-level thoughts

1. The discussion does talk about model violations, but only in terms of fitting single-season models when the data did not arise from that process. Again, given the way the study design is presented, I am uncertain about how prevalent the resampling of sites is. If there was no resampling over time, then a single season model is actually okay to use (though I would still consider blocking by year, or treating it as a random effect, as there is no doubt important yearly variation).

What the discussion does not do, however, is discuss the importance of how the sampling duration affects abundance estimates. There are a number of papers that have already shown how this binning process can result in over-estimates of local relative abundance (cited in me review and in the current manuscript draft for some, like Link et al. (2018)). To me, this calls into question how useful such models are for camera trap data. It seems that by discretizing the sampling process into sample days, weeks, etc., you often end up inflating repeat counts of the same individuals. As such, your abundance estimates go up. The discussion does not really unpack this idea, nor does it provide solid suggestions for how to possibly address this. Personally, I would imagine that much more care needs to be taken when generating your counts for modeling purposes, as using the 1-minute threshold as was done here clearly shows that problems can occur.

2. Was the hypothesis / model predictions revisited here in the discussion? If so, this was done implicitly when it should be explicit. Coming back around to them is a key way to make the paper read more cohesively.

Line by line comments

Line 614 - 615: They would first need to develop a clear way to avoid double counting across a given detection window, as this is what is causing the issues in your model. If you can do that, then it would seem to me that you would end up with identical results across models. To demonstrate this, I went back to my simulations, but kept track of individuals in order to avoid double counting if it occurred. The average bias dramatically decreased, which just goes to show how if you can limit double counting, the sampling window duration may matter less than you think. Regardless, fitting the model with varying windows may be a key way to demonstrate how well your model fits this assumption, so I still think your suggestion stands (but perhaps for a different reason than the paper suggests).

Simulation code, but tracking individuals instead in the simulation process (still the same model).

```
# try again, but keep track of individuals in order to avoid double counting
#  when going from 8 to 4.

library(nimble)
library(MCMCvis)

set.seed(565)
# number of sites
n <- 150
# number of replicates
j <- 8

# intercept only model
lambda <- log(10)

# latent count
y <- rpois(
  n,
  exp(lambda)
)
max_y <- max(y)

y_ind <- matrix(
  0,
  ncol = max_y,
```

```
  nrow = n
)
for(i in 1:n){
  z_vec <- rep(c(0,1), times = c(max_y - y[i], y[i]))
  y_ind[i,] <- sample(z_vec)
}

# low detection prob at j = 8 replicates
rho <- 0.1

y_obs_ind <- array(
  0,
  dim = c(n,j, max_y)
)


for(t in 1:j){
  for(i in 1:n){
    for(s in 1:19){
      y_obs_ind[i,t,s] <- rbinom(
        1,
        1,
        0.1 * y_ind[i,s]
      )
    }
  }
}

# get observed count from this for model.

y_obs <- apply(
  y_obs_ind,
  c(1,2),
  sum
)


my_model <- nimble::nimbleCode({
  lambda ~ dnorm(0, 2)
  rho ~ dlogis(0,1)
  for(site in 1:nsite){
    log(log_lambda[site]) <- lambda
    y_latent[site] ~ dpois(log_lambda[site])
    for(rep in 1:nrepeat){
      logit(det_prob[site,rep]) <- rho
      y_observed[site,rep] ~ dbin(
        det_prob[site,rep],
        y_latent[site]
      )
    }
  }
})

data_list <- list(
  y_observed = y_obs
)
constant_list <- list(
  nsite = n,
  nrepeat = j
)

# fit with the j = 8 repeats
model_j8 <- nimble::nimbleMCMC(
  code = my_model,
  data = data_list,
  constants = constant_list,
  monitor = c("y_latent", "rho", "lambda"),
  niter = 40000,
  nburnin = 10000,
  nchains = 3
)

j8_summary <- MCMCvis::MCMCsummary(
  model_j8,
```

```
  round = 2
)
# see if they converged (yes)
range(j8_summary[,6])

tail(j8_summary)

y_obs_j4 <- matrix(
  NA,
  ncol = 4,
  nrow = n
)
col_vec <- matrix(1:8, ncol = 4, nrow = 2)
for(i in 1:n){
for(t in 1:4){
  tmp <- colSums(
    y_obs_ind[i, col_vec[,t],]
  )
  if(any(tmp>1)){
    tmp[tmp>1] <- 1
  }
  y_obs_j4[i,t] <- sum(tmp)
}
}



data_list2 <- list(
  y_observed = y_obs_j4
)
constant_list2 <- list(
  nsite = n,
  nrepeat = ncol(y_obs_j4)
)

# fit with the j = 8 repeats
model_j4 <- nimble::nimbleMCMC(
  code = my_model,
  data = data_list2,
  constants = constant_list2,
  monitor = c("y_latent", "rho", "lambda"),
  niter = 40000,
  nburnin = 10000,
  nchains = 3
)

j4_summary <- MCMCvis::MCMCsummary(
  model_j4,
  round = 2
)
# we would expect rho to be different between models,
#  but the other variables should not especially change.

# plot estimates
to_plot <- data.frame(
  j8 = j8_summary[-c(1:2),1],
  j4 = j4_summary[-c(1:2),1]
)

# you end up with higher abundance with the four replicate
#  binning process (all points under line).
plot(
  to_plot$j8 ~ to_plot$j4,
  ylim = c(5,20),
  xlim = c(5,20),
  xlab = "Four replicates",
  ylab = "Eight replicates"
)
abline(a = 0, b = 1)
```

Tables & figures

Just a little picky, but the color squares in the bottom right of the species specific figure could be moved a bit closer to the text (strongly positive, moderately positive, etc. etc.). Likewise, I'm unsure why these figures have box and whisker plots as well as bar plots. The strongly positive color hides the box and whisker plots, so you can't see the median all that well. Not really sure what the fix here is, perhaps coloring in the box and whisker plot could help (or having points and lines for median and CI).
