# Supplementary material for: Combining N-mixture and occupancy analysis offers a more complete picture of carnivore habitat use in Northeastern Türkiye
Source: PLoS One. 2025 May 14;20(5):e0320768. doi: 10.1371/journal.pone.0320768 (PMC12077724; doi:10.1371/journal.pone.0320768)
Supplement: Attachment — Submitted filename: Response to Reviewers.docx [file pone.0320768.s008.docx]

The authors have updated the original manuscript to comply with the suggestions of the reviewers. We would first like to thank the reviewers and the editors for their patience and understanding while we re-ran all of the analysis. While re-running the analysis we were able to incorporate a new year of data and excluded locations that were near rubbing to further reduce bias.

Response to Reviewers:

Reviewer #1:

Major comments

1) Line 70. This hypothesis is not informative. Of course habitat selection must vary among wolves, bears, and lynxes (your current hypothesis is quite obvious), but you need to elaborate on how these inter-specific differences must be. What differences do you expect among these three species in habitat selection? Which landscape features likely facilitate (or prevent) both the presence and large abundance of each species? You must propose a more informative hypothesis based on the biology and ecology of each species.

Response:

We added in a section after our hypothesis predicting how each animal would react to different features.

2) Lines 129-131. A major frailty of this study is that vegetation cover (specifically, tree canopy cover) was not considered as a potentially important predictor of both occupancy and abundance of the three focal species. Several studies in different animal taxa have demonstrated that vegetation cover heavily influences whether a species is present in a particular area (and also likely its site-specific abundance). Thus, why you did not consider this variable as a potential predictor in your occupancy and abundance analyses? I certainly think that bears, wolves, and lynxes prefer sites with abundant vegetation cover. Hence, without this covariate your analyses and results are incomplete and likely misleading. Fortunately, you can obtain quantitative data on vegetation cover using geographic information systems (e.g., satellite images). You could do this for different patch areas around the location of each camera trap (e.g., circles of 0.5, 1, or 2 km in diameter). Without this covariate in your models, I cannot assess the reliability of your main findings or the validity of your conclusions.

Response:

We agree with the Reviewer. We originally used distance to forest edge as a proxy for cover. However, to respond to this comment from the reviewer, we created 3 levels of forest cover (500 m radius, 1 km radius, and 10 km radius). 1 km and 10 km radii were both highly correlated to other covariates. However, 500m was not, and was introduced into the model. Since both forest cover and distance from forest edge covariates are proxies of each other, we ran the full model (occupancy, N-Mixture) for both under all sampling windows and compared them to each other in both univariate and multivariate frameworks. In the multivariate approach, forest cover and distance to forest edge were the only covariates used. While we still believe distance to forest cover may be better suited, forest cover had stronger effect sizes in more categories, and is more prevalent in the literature. We have attached the results for the full model when we used forest cover, distance to forest edge, and both for the reviewer’s consideration.

3) Lines 144-145 and 150-154. Here, you say that you used a Poisson distribution to build your N-mixture models. Did you verify that this is the appropriate distribution for your three study species? You may also use negative binomial and/or zero-inflated Poisson distributions to build N-mixture models (see Kéry and Royle 2016, Applied Hierarchical Modeling in Ecology, Vol. 1, Academic Press). You must test the appropriateness of these distinct distributions for each focal species. In particular, you say in lines 114-116 that the lynx has the lowest densities based on limited captures on camera traps. Thus, a zero-inflated Poisson distribution could be more appropriate to model the abundance of this species.

Response:

We thank the reviewer for their response, and for raising an important issue with many ecological modeling approaches. For our model, although we our latent site abundance using a Poisson distribution, we do introduce an over dispersion parameter, which is quite similar to a negative binomial approach. Essentially, this parameter helps control for large individual differences between sites that a typical Poisson distribution would struggle with (i.e., would produce over-inflated estimates difficult to ecologically interpret). However, although our data for lynx was sparser than for that of both bear and wolf, this species was still detected at ~80% of all survey sites, which is much higher than values recommended by Kery and Royle where a zero-inflated parameter may need to be included. In fact, these high values of occurrence for each of our target species was a major motivator for our inclusion of N-mixture modeling in the first place.

4) More details are needed about the different competing models (both N-mixture and occupancy models) that you fitted to the data of all three study species. You should have compared the effects of your distinct predictor variables (and perhaps additive or interactive combinations of predictor variables) on occupancy and abundance. However, in your methods you do not explain if and how you did such comparisons among competing models with distinct environmental predictors. You mention several times in the manuscript that you got a “final model” (for both abundance and occupancy, see lines 157-158, 181-182, and 192) but you fail to explain how did you arrive to such final models.

Response: We apologize if the term “Final model” was confusing. We excluded Ruggedness and, now, distance to forest edge as covariates. This is explained in the paragraph beginning on line 197. Furthermore, while crafting our model, only two covariates, ruggedness and slope, were highly intercorrelated (Person's |r| > .7). A univariate analysis was run using each of the two covariates, and the effect of slope was greater than that of ruggedness for all species (data not shown). Therefore, slope was included and ruggedness was excluded from the final analysis.” We did not include information based on distance to forest edge as we feel it is not relevant now that we have forest cover. We have tried to clear this confusion by explaining our “final” model, and have removed that term from the manuscript.

4 cont.) Furthermore, in your results you say that “all N-mixture models had acceptable fit to the data” (line 242) and “all occupancy models also had acceptable fit” (line 284), but it is not clear, at all, which were all these N-mixture and occupancy models. I insist, you must describe clearly all your competing models and how did you select the best predictors for both abundance and occupancy.

Fit for the occupancy model is based off the Bayesian p value, and fits for the N-mixture models are based off Bayesian p-value and over dispersion is measured with c.hat values (described in line 227 “Finally, model fit was assessed using a Bayesian p-value and Ĉ statistic for the N-mixture model and a Bayesian p-value for the occupancy model. Bayesian p-values and Ĉ statistics were calculated from simulated and observed data (13,43), where Bayesian p-values between 0.25 and 0.75 indicate a good fit and a Ĉ less than 1.1 suggests minimal unaccounted overdispersion (44,45).”) These measures have been used to measure goodness of fit throughout the literature (see Kéry and Royle 2016, Applied Hierarchical Modeling in Ecology, Vol. 1, Academic Press pg.75, 250-254; Gelman et al. 2014 )

5) It is not clear where do your estimates of abundance (figures 2, 3 and 4) and occupancy come from? Did you extract these estimates from your final models for each species? Are they predicted abundances and occupancy probabilities for a site with average values of your environmental covariates? Are they model-averaged estimates (across all competing models)? Please make sure to clarify how exactly did you calculate the main abundance and occupancy estimates that you show in figures 2, 3 and 4.

Response:

We apologize that these values were unclear. In our figures, estimates of abundance and site use probability come from the final model, where predicted values are derived quantities from the joint posteriors. Each site-specific value is calculated based on the average value of each standardized covariates, and no estimates are model-averaged. This has been clarified in the captions.

Additional comments

Abstract

Line 32-33. It is not entirely true that “varying the detection window did not significantly affect modeling outcomes”. According to your results, different detection windows can yield distinct estimates of site-specific abundance of two of your studied species (bears and wolves, see figures 2 and 3). This is a critical result that must be mentioned in the abstract.

Response: This has been included in the abstract.

Introduction

Lines 41-42. You should mention as well potential effects of features of the landscape such as vegetation cover, availability of water bodies, topography, etc.

Response: The authors have decided not to implement this suggestion. This section is vague as the exact effects of the covariates mentioned by the reviewer are context dependent and may differ among species. Adding this into the paper would not add to the background needed to understand the paper. Furthermore, we go into more detail into this in a later section.

Line 50. Insert a space between “habitat selection” and “(13–15)”.

Response: Corrected as suggested

Lines 58-60. The first part of this sentence is awkward and hard to understand. What exactly do you mean by “less sensitive when species are rare and wide-ranging or common”? Are you trying to compare the sensitivity of occupancy models between rare, wide-ranging, and common species? Are occupancy models less sensitive when applied to rare species compared to when applied to common species? Is this what you want to say here? You are mentioning here rare and wide-ranging species but what about species that are rare and geographically restricted? All this is unclear, please reword to clarify.

Response:

Occupancy analysis is difficult when a species is both rare and wide ranging, or if the species is ubiquitous across the landscape. If you have all 0’s (a species that is rare and wide ranging) or all 1’s (at all sites) it is impossible to derive differences between sites. We have changed “common” to “common and ubiquitous” to better highlight this distinction.

Lines 63-64. Here you mention that there are a few studies that have compared the results of N-mixture and occupancy models, but you do not provide the references to such studies.

Response: Corrected. There are no other studies like the one presented here.

Methods

Line 108. “Lupus” must not be written with capital “L” (i.e., must read “lupus”).

Response: Corrected as suggested

Line 109. A period must be inserted before “While”.

Response: Corrected as suggested

Paragraph that starts in line 119. You must mention in this paragraph what was the minimum distance between cameras and what was the total number of cameras.

Response: 285 cameras were deployed at 150 different locations. Locations were on average 886m apart from each other. However each deployment was considered separate since rates of humans and vehicles frequently vary by season (see Blount et al. 2024). This information has been clarified in the methods and results.

Line 124. Insert a space between “3” and “m”.

Response: Corrected as suggested

Line 138. Add pertinent references to this sentence that opens this section.

Response: Corrected as suggested

You write “occupancy” throughout the manuscript using a capital “O”. I think this is incorrect and everywhere it must be written as “occupancy”.

Response: Capitalization of occupancy has been checked and corrected throughout the manuscript.

Line 145. “Binomial” must not be capitalized either.

Response: Capitalization of binomial has been checked and corrected throughout the manuscript.

Line 185. You did not define what “Zi,k” denotes in this formula. Please explicitly define this term.

Response: Z­_i,k_ is a measure of occupancy described in line 175-176. The authors went back and clarified this section

Line 199. Why are you making reference to Table 1 here? This table includes your list of environmental covariates and, in contrast, this sentence is talking about species-specific parameters. This is confusing, please clarify.

Response: This has been simplified to just “parameters”. The original purpose of “species-specific parameters” was simply to convey we were looking at how the covariates effect each species independently, but also together on the community level through the use of hyperparameters.

Lines 216-219 and elsewhere. You use both uppercase “P” and lowercase “p” to denote P-values. Please be consistent.

Response: All instances of P-value were changed to p-value. The document was scanned to ensure consistency.

Line 220. I would use the Greek symbol chi followed by a superscript 2, instead of spelling out “chi-squared”.

Response: Corrected as advised

Results

Lines 254-257. This is not entirely true. According to the credibility intervals (CIs) shown in the first panel of figure 3, the 10-day window differs significantly from most other time windows (this CI does not overlap with most other CIs). In addition, the CI range that you report in the text for the 10-day window does not match the CI that can be seen in the first panel of figure 3. In the text, the lower limit is 2.608, whereas in the figure the lower limit is a little higher than 3.5. Please make sure to correct this first panel because it is incomplete and the mean estimate for the 10-day window cannot be seen.

Response: This has been corrected in the rewriting of this section. This has been clarified within the new section.

Please make sure that in all your results you write “differences between time windows”, because in some sentences (e.g., lines 254-255 and 257-258) you write “there was no difference” but you do not specify differences between what? Thus, you must always clarify that you are talking about differences between your distinct time windows in parameter estimates.

Response: This has been corrected in the rewriting of this section. This has been clarified within the new section.

Line 257. Replace “between” with “in”.

Response: Corrected as suggested

Lines 301-312. In this paragraph, where you present community effects, you make reference to supplementary Tables S1-S3, but the numerical results from your community effects are in Table S4. Please explain or correct this discrepancy.

Response: Corrected as suggested

Line 309. I think “besides” must be replaced with “except for”.

Response: Corrected as suggested

Line 311. Do you mean “survey effort”? In your methods, you described this variable as “effort” (or “survey effort”). Thus, I would use this terminology all throughout the manuscript to avoid confusion. In addition, this variable only affected detection probability, right? (as I mentioned before, you did not describe clearly your candidate models, how you obtained a “final model”, and which covariates were included in this “final model”). If this is the case, you should mention here explicitly that this significant positive effect of survey effort occurred only on detection probabilities.

Response: This has been removed in order to increase clarity.

Line 322. Reword as: “The average mean effect of survey effort on detection probabilities was” (assuming that survey effort only affected detection probabilities, right?).

Response: This has been removed in order to increase clarity.

Lines 345-348. Was human activity really associated positively with occupancy of bears? This is what you say in this text, but in both Table S1 and figure 5 the effect of human activity on occupancy is NEGATIVE in all detection windows. In fact, the average mean effect of human activity that you report in this sentence is -0.18 (line 346). Am I missing something here? Please correct or clarify.

Response: This comment is no longer applicable due to re-running the analysis and re-writing this section.

Line 372. Why are you making reference here to figure 3? Shouldn’t this be figure 6 instead?

Response: Corrected.

Lines 394-396. How come the average mean effect of human activity on lynx occupancy was positive (0.14), if all the individual effects (for each time window) of this predictor variable were negative (see figure 7 and Table S3)? Is this a mistake? Please correct or clarify.

Response: This comment is no longer applicable due to re-running the analysis and re-writing this section.

Lines 414-415. Here, you must emphasize again that, contrary to site occupancy, the expected counts of bears and wolves did vary among detection windows. This is a critical result.

Response: This has been incorporated into this section.

Discussion

Line 420. Please provide again here the main citations to the N-mixture and occupancy models.

Response: Corrected as advised

Line 429. Reword as “abundance. Therefore, these three aspects…”

Response: Corrected as suggested

Line 442. Insert a period between “(47-49)” and “Furthermore”.

Response: Corrected as suggested

Lines 449-451. Please remind to the reader here that occupancy models detected fewer environmental effects than N-mixture models.

Response: corrected as suggested

Line 478. Please make sure to emphasize in this sentence that the effect of vehicles on bears was POSITIVE.

Response: This comment is no longer applicable due vehicle effect being removed.

Lines 486-488. These two sentences sound redundant. In both you say that wolves prefer sites farther from the dump and with more vehicle activity. What is the difference between these two sentences? My guess is that in the first one, you are talking exclusively about site occupancy (presence only), whereas in the second you want to say that also in these types of sites (farther from the dump and with more vehicle activity) their abundance is higher. Please reword this text to make this distinction clearer.

Response: This has been corrected in the rewrite of this section

Line 491. What is “95% aKDE”?

This has been corrected to include the entire name. aKDE is a way to estimate of Home range size. This has been simplified in the paper and a new citation was added.

Line 493. “Rendezvous” is an uncommon term. Please try to use a more common term.

Response: This is a very specific term that refers to an important location that occurs within a certain time of the year for a wolf pack. To simplify this, I would use “vital area” like was done in the sentence.

Lines 493-495 and 503-504. Here you are actually highlighting the importance of vegetation cover for wolves and lynxes. Hence, I insist that you must include this variable in your analyses. Otherwise, your ecological story is largely incomplete.

Response: We have included forest cover as a covariate.

Line 502. “More forest interior” is confusing. Do you mean that lynxes prefer sites FAR from the forest interior or DEEP INSIDE the forest interior? Please reword to make this clear.

Response: This comment is no longer applicable as we changed “distance from forest edge” to “forest cover” as suggested by this reviewer.

Line 511. Replace “dominate” with “dominant”.

Response: Corrected as suggested

Lines 514-515. One important effect of distinct detection windows occurred on the estimated abundances of bears and wolves. I think that is an important result that must be mentioned in this paragraph. Your first sentence in this paragraph minimizes the importance of this effect of detection windows on estimated counts.

Response: This has been added in.

Line 529. Replace “counts” with “parameters”.

Response: Corrected as suggested.

Conclusions

Lines 535-537. This conclusion is partially wrong because distinct detection windows did have important effects on expected counts of bears and wolves. Please modify it accordingly.

Response: Corrected as advised.

Tables and figures

Line 134. “Occupancy” must not be capitalized.

Response: Corrected as suggested. The rest of the document was scanned to ensure occupancy was not incorrectly capitalized in other locations.

Table 1. Define what “ESRI” and “CLC” stand for, either in the table heading or as a foot note.

Response: ESRI is a company, CLC has been defined as suggested.

Table 1. Are you sure that “Digital Terrain”, “Model”, and “Camera” must be capitalized? I don’t think so. Please verify.

Response: Corrected as suggested.

In all your figures you must explain (in the figure captions) what do the error bars denote. Do they represent credibility intervals (CIs)? If so, are they 95% or 85% CIs?

Response: Corrected as suggested.

Captions of figures 2, 3, and 4. Reword as follows: “differences among time windows in N-mixture expected counts, site use probability (occupancy), and detection for…”

Response: Corrected as suggested.

Figures 2, 3 and 4. Do not spell out “lambda”. Use instead the Greek symbol lambda.

Response: Corrected as suggested

Figure 3. The first panel is incomplete. The mean estimate of expected counts for the 10-day window cannot be seen.

Response: This has been explained in the caption.

Captions of figures 5, 6, and 7. Please explain in these captions what do the error bars denote (both thicker and thinner error bars).

Response: Corrected as suggested.

Reviewer 2

**Primary Issues**

*1. Methods section (including explanation of occupancy and n-mixture modelling covariates and modelling process, and provision of additional information)*

I believe the Methods section requires some reworking. I feel there are a few shortcomings in the way the methodology is presented and discussed. A lot of information (more than it usually is) is given about certain aspects of the methodology; this is fine, and not necessarily an issue. However, virtually none is given about some other aspects, some of which are arguably even more important. These include:

a) Model fitting: The authors go into great detail on how the models were fit. However, they don’t say anything about the step just prior to that. How many site covariates were included in each model? Did they only fit univariate models, or multivariate models? If the latter, how many covariates in each model? How did they determine this? As with occupancy there is a risk of overparametrisation when fitting models, which can lead to misleading beta coefficient results, it’s important to clarify this.

Response: This has been included in the “Covariate selection” section of the paper. We included all of the covariates we believed had a plausible effect of habitat use from similar studies in other locations, as well as our knowledge on the ground. Only covariates that were highly correlated were removed as discussed in the covariate selection section. In order to assess goodness of fit and to reduce overparameterization, we used a Bayesian p-value.

b) Detection covariates: Were detection covariates used? For example, the authors mention that some cameras were located on roads, due to large carnivores’ tendency to use these to travel. Were some cameras not on roads, and if so, was road placement included as a detection covariate? Were any other detection covariates used? Ideally, the authors would have done so; if they did not, I would expect at least a mention of detection covariates, however.

Response: All cameras were located on roads and were placed in the canopy. We did this so we would not have to account for differences in placement or height. Only survey length, which was log transformed was used as a detection covariate.

c) Size of sampling site and extraction of covariates: The authors mention the study area was divided into 2 km2 “grid cells”. Did these grid cells act as the “sites” for the modelling? And were covariates extracted at the scale of these? I think needs to be clarified in text.

Response: The 2x2 grid was only created to force researchers to place cameras throughout the study area, while reducing overlap between cameras. This has been included in the “camera trap assay” section.

d) Survey length: The authors mention cameras were left in the field for an average period of 81 days. Is this per year? If so, it needs to be clarified.

Response: This has been clarified in the paper. The cameras were left on average for 81 days before being moved to another site.

e) Information on covariate justification and calculation: I think it’s good practice to include information on why covariates were included. As the authors have already done most of the work for this by including Table 1, I would recommend changing “Description” to “Description/justification”, and adding for each a line about why the covariate is expected to potentially influence habitat use of the target species. Similarly, I think more information is needed on how each covariate was calculated, not just from what source. E.g. How was vehicle activity measured? Vehicle per days? Same for some of the other covariates. This doesn’t necessarily have to go in the main text, it can be supplementary material, but I do think it’s important to include it.

Response: A section called “covariate selection” has been added to the methods and includes justification and scales. It also further describes how vehicles and human presence is measured.

f) Model ranking: No information is provided on the model ranking process. Which models were retained and used to interpret covariate coefficients following model rankings? For each species, are the coefficients presented from the top model, the most parametrised model with strong support, etc.? Again, more information needs to be given on this.

Response: the authors included clarification for this in the covariate selection process. We used all non-correlated covariates in our model. The Bayesian framework does not provide for a good way to do model selection. Furthermore, all of the covariates were highly plausible to have an effect on habitat use.

Overall, I think more clarity is required on all the above.

*2. Interpretation of results and of differences between occupancy and n-mixture outputs*

I am not convinced by some of the interpretation of the outputs from the occupancy and n-mixture modelling.

Firstly, the authors interpret some differences in results from the two modelling approaches as being a result of differences in abundances between the different species. For example, they state that “We found that N-mixture results were more sensitive for all species, but increasingly so as a species’ abundance increased”. How do they know that the reason for n-mixture modelling resulting in more covariates being significant (what I understand “more sensitive” refers to) is a result of differences in abundance between species, and not a consequence of some species having their local abundance being more strongly driven by environmental factors than others? I feel the authors are making some important, untested assumptions with their interpretations of results.

Response: We removed the line “ but increasingly so as a species’ abundance increased.

Similarly, the authors state that “Finally, when analyzing habitat selection at the local population level, we recommend using a combination of N-mixture and occupancy modeling, as they may be able to provide a better understanding of species’ habitat use. At a coarse scale, occupancy analysis can reveal habitat selection for the entire population, which may provide a better understanding of what limits a population’s range, while at a finer scale, N-mixture modeling offers more detailed insight into how intensely used certain habitats are within a species’ range”. I don’t think, however, that this is question of “scale”. As the authors themselves state, occupancy can be used to investigate factors affecting presence-absence, while n-mixture factors affecting relative abundance. That’s the difference – not one of scale. I understand what I believe is their underlying point – that occupancy works better at larger spatial scales, due to this being 1/0, while n-mixture modelling *could* be more effective at finer spatial scales due to its ability to model count data, and therefore identify differences in habitat use that occupancy cannot – but as it stands the authors are making comparisons about scale that are not backed up by their study, both modelling approaches were applied to the same spatial scale (as sampling sites were the same). I would therefore strongly recommend shifting the focus from *scale* to discussing how both methods can be

used to gain complementary insights into a population, in the form of how factors affect occurrence *and* relative abundance of a species in the study area.

Response: The conclusion has been shifted to incorporate the guidance of this reviewer.

**Additional minor issues & comments**

**Across:** The authors use “habitat use” and “habitat selection” interchangeably throughout, and discuss how occupancy and n-mixture modelling can be used to study habitat selection of target species. However, I don’t think this is correct – any HSM tool, as occupancy is, gives information on habitat *use*, not *selection*, as the latter implies a preference factor which we do not know is necessarily there. It’s a small distinction, but I think an important one. I would therefore suggest the authors use “habitat use” only throughout the manuscript if referring to the observed occurrence-covariate relationships.

Response: Corrected as suggested throughout the manuscript. Thank you

**Fig. 1** – A bit more detail on the legend. What’s the green? Forest?

Response: Corrected as suggested.

**111** – I think you need to caution your statements about the relative abundance and density of each species a bit better. You do not have comparable density or abundance estimate; I don’t doubt there are more bears and wolves than lynxes, but you state that bears are “far more abundant” than wolves, yet you present two metrics that are different. Again, I’m not saying that’s not necessarily the case, but I would rephrase this section to acknowledge limitation and better justify your statements.

Response: I have included more information to help clarify this statement. There are roughly 28-30 wolves most years, however the minimum number of bears seen at one time in the system is 41 and genetic based data sets the minimum above 90. This has been added to the paper.

**121** – A bit unclear; what was randomly selected?

Response: This has been clarified in the text.

**124** – 81 days in what? Per year every year? Or overall over 11 years?

Response: This has been clarified in the text.

**129** – Covariate choice should have its own sub-section, separate from the field survey/data collection. Also, at what scale were they extracted? What was the site size? See major comment above

Response: Covariate selection has been included as a sub-section.

**141** – Don’t capitalize “Occupancy”

Response: Corrected as suggested. The rest of the document was scanned to ensure occupancy was not incorrectly capitalized in other locations.

**148-149** – Unclear what this means

Response: The authors are uncertain of what this comment is meant to include. Therefore we will address the two sentences in this line. In the first sentence we are describing what type of model we chose, and how we modified it. This is later discussed in the methods. The second sentence addresses an important point of how we control for overdispersion which was brought up by the other reviewer.

“For N-mixture models, we modified a Bayesian co-abundance N-mixture model (12) into a multi-species, multi-year model.”

**191** – Why is this brought up here, and why is it mentioned here even though it’s relevant to both n-mixture and occupancy?

Response: This has been moved to an earlier section describing covariates.

**317** – Vehicle activity; how was this done? Were all cameras on road? If not, include detection covariate? Again, see major comment above.

Response: this has now been described in the Covariate selection section.

**409**: “Overall, the N-mixture model was more sensitive to factors affecting fine-scale habitat usage” What does this mean? We haven’t introduced the ideas of scale? See above – I’m not convinced you can speak about spatial scales with your results.

Response: We re-evaluated all instances where “scale” was used. While it is still included in some situations, it is specified that occupancy looks at the boundaries of the study area, while N-mixture can tease out relative abundance within those boundaries.

**418**: Again, please caveat that lower abundance is an assumption, and not something confirmed. Otherwise, if basing it on camera trap captures, I would suggest saying “relative abundance”.

Response: The authors have qualified this statement

**436**: “Total abundance was lower” – again, making some statements that I simply don’t think you have the data to make, even if this may be the case! I would rephrase to acknowledge this.

Response: Since we clarified population estimates earlier in the paper, we have left this in.

**442**: Full stop missing

Response: corrected as suggested.

**460 – 462**: Again, see major point above – I’m not sure it’s a question of scale; at least, this is not what you are testing. I would rephrase as suggested above.

Response: Corrected as suggested.

**500**: “or that these species react similarly to both small and large-scale changes on the landscape.” Same point

Response: Corrected.

**553**: “We found that N-mixture results were more sensitive for all species, but increasingly so as a species’ abundance increased” See major point above – not convinced by this interpretation.

Response: this was removed

**Fig. 5** – Can’t really see much here.

Response: All three graphs have been redone and had their size increased.
