# Supplementary material for: Combining N-mixture and occupancy analysis offers a more complete picture of carnivore habitat use in Northeastern Türkiye
Source: PLoS One. 2025 May 14;20(5):e0320768. doi: 10.1371/journal.pone.0320768 (PMC12077724; doi:10.1371/journal.pone.0320768)
Supplement: Attachment — Submitted filename: Response to reviewer_Mason.docx [file pone.0320768.s009.docx]

Response to reviewer:

The authors would like to thank this reviewer for one of the most in depth and well thought out reviews we have seen. We consider the paper much stronger after following the guidance of this reviewer. This reviewer had three major issues with the stats used in this paper: changes in detection window “binning” animals creating inflated counts, double counting from independent detection windows, and a lack of control for years within the models. We believe that we have successfully addressed all three of these major issues and the numerous minor issues brought as well. Specifically, we withdrew the consideration of detection windows from this project. We believed that while it is still important to consider, it went beyond the scope of this paper, and would require extensive simulation studies. The authors are excited to potentially address this issue in a future paper. To reduce double counting from independent detections, the authors went through every detection and shifted their windows to limit if not eliminate double counting of the same individual. Finally, we controlled year by allowing the detection probability to change between replicates and capped the number of replicates at each site to 4. We also added all of the reviewer’s suggestions and cleaned many parts of the manuscript for better clarity. Below we have addressed our response to each individual point.

1. There are still some issues identified by the previous reviewers that exist in this draft. I, for example, had a lot of trouble determining what covariates were used within each model given Table 1 and the 'covariate selection' section. That specific issue is not insurmountable, but readers should be able to clearly and easily understand the model fitting procedure, which includes which covariates were used where.

Response: we added a new section in Table 1 that outlines what covariates were used in the final model. We hope by clarifying sections in the methods and including a new section in the table, this is now easier to understand.

2. The paper lacks any justification for why varying detection windows are used, and why we would want to compare these across models. I think this comparison could be useful, but the paper needs to set the foundation for why this matters, and at the moment I feel like this responsibility is being left with the reader (which is not ideal).

Response: We ended up removing the sections about varying detection window. While we think it is still an important topic, we understand that to fully answer this question would draw away from what we feel is most important in this paper, comparing N-mixture and occupancy results. This has resolved many of the issues that have been brought up in this review.

3. This is related to the previous comment, but I have moved this to a different section there is a bit to unpack here. Basically, I'm left wondering why a shift in detection probability, which comes with a decrease in the number of secondary surveys that could occur, results in an increase in counts from the N-mixture model. I'm assuming that the binning process here results in greater counts in the observed data, which may be causing this? This, to me, indicates a rather glaring violation of an assumption for this class of model, which is that counts are unique (i.e., an individual is not double counted).

Response: To resolve this issue, the authors went through camera trap phots to assess independence between photos and time for the same individual to be photographed on two occasions. This has been covered in greater detail in the results section of the paper and has been independently discussed with the reviewer.

Abstract

1. The distinction that the introduction makes between occupancy models and n-mixture models, especially as applied to camera trap data, is perhaps not as distinct at the paper tries to make it. The observational level of the detection model can still be interpreted as intensity of use (e.g., a species is detected at one site on two surveys while at another site of 10 surveys). This can be especially helpful when a species occupies close to the entire landscape (e.g., Gallo et al. 2018).

Response: This has been added into the introduction.

2. There really is not much justification why the repeat sampling window duration was modified. There has been some very recent research that has looked into this as a way for accounting for temporal autocorrelation in a given detection history (Goldstein et al. 2024).

Response: Repeat sampling duration has been removed from the paper.

3. The hypothesis is one that would be pretty hard to disprove, which is something the reviewer in the last version of this manuscript pointed out. I think that predictions were provided that help flush this hypothesis out more, but the hypothesis on it's own still needs improvement. Species should be expected to vary in habitat use, modeling different types of data with different link functions should result in different results. If you want to use a hypothesis here, then I would instead try to focus on the comparison. How do you think occupancy and n-mixture models compare? Do you think one will outperform another? Will that relationship vary based on the ecology of a species? Data density? It's 100% okay for a hypothesis to be wrong, but in it's current form this specific hypothesis is not all that falsifiable given that the null would here is 'species use habitat the same way and these models work the same' which we don't need this study for to pretty reliably reject.

In fact, do you really need a hypothesis here? You've already stated two major questions (line 84 - 85), why not just focus on answering those?

Response: We removed the hypothesis here and focused on answering questions as advised by the reviewer.

Line by line comments

line 62: repeat-sampling observational data is perhaps not the most appropriate phrase to capture what is meant here. Maybe instead this sentence could be something like: For example, occupancy (10) and N-mixture models (11-13) are two commonly used statistical frameworks that can quantify habitat use while also accounting for the vagaries of sampling (e.g., imperfect detection). or some derivative of this. No need to get into the camera trap side of things yet in my opinion, plenty of time for that later.

Response: Corrected as advised

Line 73 - 77: If you want to combine the ecological description of the problem with a statistical description, you could indicate that when species are rare or common occupancy models have boundary estimation issues (i.e., trying to estimate probabilities close to 0 or 1). Clipp et al. (2021) talk about this problem in the case of multi-species models, but the idea generally applies.

Response: Corrected as advised, citation added.

Line 79 - 80: Here are two papers that have applied both types of models to the same data set.

Response: new citations to these papers were added and the statement was qualified to represent this.

Methods

Top-level thoughts

1. In the review response the authors posit that 'The Bayesian framework does not provide for a good way to do model selection.' Given the large number of papers that use information theoretic approaches to compare Bayesian models and ecology papers that have assessed how Bayesian model selection applies to latent state models, this response from the authors is not that accurate. Certainly, Bayesian model selection is a bit more tricky as you often have to code up the likelihood yourself, but it is still something that can be done. I'd encourage the authors to check out Hooten and Hobbs (2015), which has a lot of great information on Bayesian model selection, as well as Broms et al. (2016). I've used various kinds of Bayesian model selection in my own research with occupancy models, and data code for those papers are publicly available (I provide a link below with the most relevant example).

Is model selection necessary here? I'm not sure. Right now, based on the methods, I am left uncertain what covariates were used in the latent state part of each model. I think the reason for this it is a bit difficult to figure that out as readers must refer to table 1 and then remove slope and ruggedness, and perhaps others as they are reported in the covariate selection part of the methods. This part of the model should be crystal clear. Easiest fix would be to add one more column to table 1 related to whether or not a given covariate was used (and please change the title to table 1 as not all of these covariates are used it seems).

Response: “The Bayesian framework does not provide for a good way to do model selection” was included in the notes to reviewers and does not appear in the paper. We agree this was a drastic oversimplification. In reality, we are interested in the effect all of these covariates have on the model, and wish to include them as they may vary between species. We appreciate the reviewers’ references and have familiarized ourself with them for future use. We have again clarified table one title and table itself and the section on covariate selection.

2. The mathematical notation here could be improved a bit. A lot of this is not using italics or bold when needed, misuse of capitalization, using * to represent multiplication, and incorrect matrix algebra. Methe et al. (2019) is a wonderful resource for this. I've pointed out issues as I've noticed them in the line by line comments here, but I encourage the authors to give the math another close look just in case I missed anything.

Response: The authors have redone the mathematical notation to reflect the corrections of the reviewer.

3. Occupancy models inherently account for number of sample days in their likelihood and therefore it is redundant to include number of active days as a covariate in the analysis. If one site has 4 days of sampling and another has 40, the detection-level of the model would use 4 Bernoulli trials for the former and 40 for the latter (i.e., you have more coin flips in which to detect the species with 40 sampling days, you don't need to include the 40 as a covariate). For example, why would sampling for 40 days increase your detection probability on the first day? Your model indicates that it could, and I cannot think of a reason why that would increase the daily detection probability. Again, assuming a 0.05 daily detection probability, the probability of detecting a species at least once over 4 days of sampling is 1 - (1-0.05)^4 = 0.185 while for 40 days it is 1 - (1-0.05)^40 = 0.87. The increase in detection probability should be from the increased number of trails, but the number of trials that occurs has no bearing on the survey-specific detection probabilities. As a simple example, flipping a coin 5 vs 10 times does not increase the probability of getting a heads on each trial. With 10 flips you have a higher probability of getting heads at least once, but again the number of flips has no bearing on the individual probabilities.

Response: We have removed survey days from the detection probability. We appreciate the clarity at which the author has made this point, both in this review.

4. I would still encourage using some covariates that may capture variation in local abundance in the detection part of the occupancy model. Local abundance does, after all, influence detection probability (McCarthy et al. 2013).

Response: After deliberation with the authors, we decided not to include site level covariates but instead allow the intercept to vary by replicate.

5. I think it would help to remind the reader that sites are not sampled consistently throughout the entire length of the study, which is why you did not account for temporal autocorrelation in local site use intensity or occurrence in your models. Right now, I could see people reading this and thinking that this is the standard way to model these relationships, and in fact it is only applicable to this specific use case. It does make me wonder though how often sites are repeatedly sampled across successive years.

Response: To reduce autocorrelation, but include as much data as possible, we thinned our data so any one site had no more than 4 replicates. This has been further explained in the methods.

6. To build off the last comment. Since there are 13 years of data, it is strange to me that neither model has a subscript related to time. This makes things a bit confusing, as what happens if a site is sampled more than once over this 13 year period? Is it treated as a new site? How does this affect the definition of your site i subscript? Some additional clarity here would be helpful as I am a bit puzzled with how your data connects to these models.

Response: we have now included an intercept for replicate. We tried to have year as a fixed and random effect and had issues with both model converging even when running at 2,000,000 iterations.

7. What are your priors for intercept and slope terms in your multi-species model? With 3 species, having species- level random intercepts and slopes is a little difficult, though you could simply just give them all independent priors (i.e., not estimate a community level average).

Response: We decided to keep hyperpriors and a community level estimate for our population as the community level effects is of interest in a future paper.

NOTE: line 265 - 269 details this. While you can fit a standard multi-species model here, I would be very careful with trying to make inference on any community level averages or the standard deviation term (see Gomes 2022).

Response: We do not make any inferences on community level averages or the standard deviation term.

Line by line comments

Line 158: Why exclude data when there were fewer than 7 active days? Those sites still provide information and the detection probability there is partially informed by sites that share similar habitat characteristics (so long as they are included in the detection part of the model). I've also subset data like this in the past, but have since realized that this is not necessary and is a bit arbitrary. Why 7 days and not 8? Why not 14 days? Excluding a bear rubbing tree

makes sense though.

Response: We have included all data points. This is now reflected in the methods and results section.

Line 170: In the review response there was a lot of focus on using canopy cover rather than distance to forest edge.

Table 1 has the latter but not the former.

Response: This has been corrected

Line 191 - 192: May want to be explicit about what you mean by detection rate here (e.g., was it the number of secondary surveys detected / total number of secondary surveys or number of independent photos / total number of days sampled). The former is bound between 0 and 1 whereas the latter would not be, so some specificity here would be nice.

Response: This was removed as we no longer included survey as a covariate on the model.

Line 221: N_{i,k} should be lowercase and italic (here and throughout).

Response: Corrected as advised

Line 223: \lambda_{i,k} should be italic (here and throughout).

Response: Corrected as advised

Line 221: define your subscripts and their range. For i in 1,...,I sites and k in 1,...,K species...

Response: Corrected as advised

Line 223: k subscript should be italic (...species k detected...)

Response: Corrected as advised

Line 225: italics for \alpha terms. Furthermore, you can just simplify this to: where a_k is a vector of species-specific parameters and x_i is a conformable vector of site-level covariates where the first element is a 1 to account for the model intercept.

Right now it looks like you are multiplying a scalar (a_covs,k) by a matrix (capital X), which is confusing. Likewise, * does not represent multiplication (though there are plenty of ecology papers that treat it as such).

Response: Corrected as advised

Line 230: uppercase Y should be y (and italics).

Response: Corrected as advised

Line 232: change P to \rho (looks like a little p) and make it italic. Uppercase P is often reserved for the probability of some occurrence / event (i.e., P(event)).

Response: Corrected as advised

Line 233: italics to subscripts.

Response: Corrected as advised

Line 235: Logit is lowercase. \beta and \epsilon should be italicized.

Response: Corrected as advised

Line 239: Missing Normal before your distribution.

Response: Corrected as advised

Line 240 - 241: This is false (see above).

Response: This was removed

Line 246: Z should be lowercase and in italics.

Response: Corrected as advised

Line 248: \psi should be italicized.

Response: Corrected as advised

Line 250: see comments above, they apply here as well. Also, you've used these terms before (\alpha and x). They are not shared among models, so either remind the reader about how while the notation is similar the parameters are not shared or just use different symbols / letters. I prefer the latter so that it cannot be misinterpreted.

Response: These were changed to 𝜑 and 𝛾 to avoid confusion

Line 258: Y should be lowercase and italics. And again, y means different things across models so this is inviting confusion. Finally, see comment about asterisk above (in fact, just drop it, p_{ijk}z{ik} already represents the product).

Response: Corrected as advised

Line 275: how long was the burn-in? Reading that sentence makes me think the burn-in was 2 steps.

Response: burn in and adaptation were the same size. For N-mixture it was 100,000 and for Occupancy it was 200,000. This has been clarified in the model fit and assessment section.

Results

Top-level thoughts

1. Again, detection probability must increase as the sampling window increases (i.e., the sampling duration).

Response: The authors have removed varying detection window from the analysis which resolves this issue.

2. I'm uncertain why inference is being made across all different sampling duration models. This is not really explained that well in the paper, and it leads to a lot of uncertainty in the results about how to unpack all these relationships. What do you do, for example, when the 10-day model finds 'significant effects' whereas the others do not? Furthermore, does average mean effect, as reported in lines 413 - 422 for example, represent the average across sampling duration models? This is unclear. If this is the case, I would caution against doing so.

Response: The authors have removed varying detection window from the analysis which resolves this issue.

3. It's a bit easier to have commas separate the values in your credible interval, as the double negatives get a little weird. Take for example, line 438 ...effect: -0.34, range: -0.43 - -0.27; mean SD: 0.21, ...I would separate all these values with semi-colons, and then use commas within a element in the list as needed. ...effect: -0.34; range: -0.43, -0.27; mean SD: 0.21;

Response: Corrected as suggested. This along with the next suggestion have been integrated and values have been reduced.

4. The species level effects have an incredibly long list of values reported for them, and it makes it very difficult to read and compare. I'm not 100% sure what the answer is here, but I had a very difficult time trying to connect the results listed there back to the species themselves. Perhaps if there was some reasoning as to what all these values need to be reported, that would help, otherwise it may be simpler to move those to a supplementary table and focus in on the key results instead.

Response: We only reported values for significant effects and limited the values we report to the means. Everything else is in the supplamnetal. We think this has significantly cut down on the clutter and mental strain of piecing these together.

Line by line comments

Line 384 - 385: If you are going to provide evidence of an effect this way, add it to the methods as well instead of right here.

Response: We removed PoE from the results.

Line 490 - 492: count data is much more rich in terms of information than binary data, so that is not too surprising (but good to see here this occur in the results).

Response: We agree. We were not surprised, but wanted to show that it occurs outside of simulated environments.

Discussion

Top-level thoughts

1. The discussion does talk about model violations, but only in terms of fitting single-season models when the data did not arise from that process. Again, given the way the study design is presented, I am uncertain about how prevalent the resampling of sites is. If there was no resampling over time, then a single season model is actually okay to use (though I would still consider blocking by year, or treating it as a random effect, as there is no doubt important yearly variation).

Response: This suggestion has ramifications that been implemented throughout the paper. In order to respond to this, we limited the number of times a site could be resampled to 4. This let us keep a majority of the data and limit spatial autocorrelation. We tried to block by year but the model didn’t converge. We also had issues with convergence when we added year as a random effect. In the end, we were able to have year replicate specific intercepts in our observation section of the model.

What the discussion does not do, however, is discuss the importance of how the sampling duration affects abundance estimates. There are a number of papers that have already shown how this binning process can result in over-estimates of local relative abundance (cited in me review and in the current manuscript draft for some, like Link et al. (2018)). To me, this calls into question how useful such models are for camera trap data. It seems that by discretizing the sampling process into sample days, weeks, etc., you often end up inflating repeat counts of the same individuals. As such, your abundance estimates go up. The discussion does not really unpack this idea, nor does it provide solid suggestions for how to possibly address this. Personally, I would imagine that much more care needs to be taken when generating your counts for modeling purposes, as using the 1-minute threshold as was done here clearly shows that problems can occur.

Response: we have removed the section of detection windows and have address the overcounting aspect in other parts of this review.

2. Was the hypothesis / model predictions revisited here in the discussion? If so, this was done implicitly when it should be explicit. Coming back around to them is a key way to make the paper read more cohesively.

Response: Yes, The first two lines of the discussion readdress our questions. We then answer them in the discussion. We have moved the discussion around to better reflect this and to cut out unnecessary portions. We have clarified this in the discussion section.

Line by line comments

Line 614 - 615: They would first need to develop a clear way to avoid double counting across a given detection window, as this is what is causing the issues in your model. If you can do that, then it would seem to me that you would end up with identical results across models. To demonstrate this, I went back to my simulations, but kept track of individuals in order to avoid double counting if it occurred. The average bias dramatically decreased, which just goes to show how if you can limit double counting, the sampling window duration may matter less than you think. Regardless, fitting the model with varying windows may be a key way to demonstrate how well your model fits this assumption, so I still think your suggestion stands

Response: The reviewer’s dedication to this point is incredible, both in effort and the clarity it provided. We appreciate the visualization of our errors, and spent quite a while addressing these concerns. We removed sampling window and went through all detections within 30 minutes of each other to ensure this bias was eliminated as much as possible.

Tables & figures

Just a little picky, but the color squares in the bottom right of the species specific figure could be moved a bit closer to the text (strongly positive, moderately positive, etc. etc.). Likewise, I'm unsure why these figures have box and whisker plots as well as bar plots. The strongly positive color hides the box and whisker plots, so you can't see the median all that well. Not really sure what the fix here is, perhaps coloring in the box and whisker plot could help (or having points and lines for median and CI).

Response: We used a combination of bar and box plots to better show the mean, SD, and CI of each model, as well as having a consistent coloring throughout the plots. We have altered the color of the dark blue to be more contrasting with the black. After spending too much time trying to shift the text in the legend closer to the significance colors, we were unable to accomplish this request. It bugs us too. We will try to manually shift these over using illustrator once the paper is accepted for publication.
