# Supplementary material for: Combining N-mixture and occupancy analysis offers a more complete picture of carnivore habitat use in Northeastern Türkiye
Source: PLoS One. 2025 May 14;20(5):e0320768. doi: 10.1371/journal.pone.0320768 (PMC12077724; doi:10.1371/journal.pone.0320768)
Supplement: Attachment — Submitted filename: Reviewer Comments.pdf [file pone.0320768.s005.pdf]

Combining N-mixture and Occupancy Analysis Offers a More Complete Picture of Carnivore Habitat Selection in Northeastern Türkiye

PLOS ONE

Reviewer Comments

Summary

Occupancy and n-mixture modelling are relatively widespread approaches to studying mammal (including large carnivore) habitat use. However, few comparisons have been carried out between these two similar yet distinct methodologies, and there is uncertainty regarding how they compare and contrast in describing habitat use. Motivated by this, the authors applied both occupancy and n-mixture modelling to a long-term camera trap dataset from Turkey. They applied these techniques to data on bear, wolves, and lynx, and discussed the model outputs in the context of what they reveal about the application and interpretation of outputs of these two methods.

Given the increased popularity of these approaches, and the aforementioned uncertainties, carrying out such a study is timely and important. Overall, the study has a strong base to build upon, and I believe will make an interesting and noteworthy manuscript. However, there are a few issues which I believe must be addressed by the authors before this is the case. Luckily, I don't think any of them should be too big of a hurdle. Below I elaborate.

Primary Issues

1. *Methods section (including explanation of occupancy and n-mixture modelling covariates and modelling process, and provision of additional information)*

I believe the Methods section requires some reworking. I feel there are a few shortcomings in the way the methodology is presented and discussed. A lot of information (more than it usually is) is given about certain aspects of the methodology; this is fine, and not necessarily an issue. However, virtually none is given about some other aspects, some of which are arguably even more important. These include:

a) Model fitting: The authors go into great detail on how the models were fit. However, they don't say anything about the step just prior to that. How many site covariates were included in each model? Did they only fit univariate models, or multivariate models? If the latter, how many covariates in each model? How did they determine this? As with occupancy there is a risk of overparametrisation when fitting models, which can lead to misleading beta coefficient results, it's important to clarify this.

b) Detection covariates: Were detection covariates used? For example, the authors mention that some cameras were located on roads, due to large carnivores' tendency to use these to travel. Were some cameras not on roads, and if so, was road placement included as a detection covariate? Were any other detection covariates used? Ideally, the authors would have done so; if they did not, I would expect at least a mention of detection covariates, however.

c) Size of sampling site and extraction of covariates: The authors mention the study area was divided into 2 km2 "grid cells". Did these grid cells act as the "sites" for the modelling? And were covariates extracted at the scale of these? I think needs to be clarified in text.

d) Survey length: The authors mention cameras were left in the field for an average period of 81 days. Is this per year? If so, it needs to be clarified.

e) Information on covariate justification and calculation: I think it's good practice to include information on why covariates were included. As the authors have already done most of the work for this by including Table 1, I would recommend changing "Description" to "Description/justification", and adding for each a line about why the covariate is expected to potentially influence habitat use of the target species. Similarly, I think more information is needed on how each covariate was calculated, not just from what source. E.g. How was vehicle activity measured? Vehicle per days? Same for some of the other covariates. This doesn't necessarily have to go in the main text, it can be supplementary material, but I do think it's important to include it.

f) Model ranking: No information is provided on the model ranking process. Which models were retained and used to interpret covariate coefficients following model rankings? For each species, are the coefficients presented from the top model, the most parametrised model with strong support, etc.? Again, more information needs to be given on this.

Overall, I think more clarity is required on all the above.

2. *Interpretation of results and of differences between occupancy and n-mixture outputs*

I am not convinced by some of the interpretation of the outputs from the occupancy and n-mixture modelling.

Firstly, the author interpret some differences in results from the two modelling approaches as being a result of differences in abundances between the different species. For example, they state that "We found that N-mixture results were more sensitive for all species, but increasingly so as a species' abundance increased". How do they know that the reason for n-mixture modelling resulting in more covariates being significant (what I understand "more sensitive" refers to) is a result of differences in abundance between species, and not a consequence of some species having their local abundance being more strongly driven by environmental factors than others? I feel the authors are making some important, untested assumptions with their interpretations of results.

Similarly, the authors state that "Finally, when analyzing habitat selection at the local 542 population level, we recommend using a combination of N-mixture and occupancy modeling, as they 543 may be able to provide a better understanding of species' habitat use. At a coarse scale, occupancy 544 analysis can reveal habitat selection for the entire population, which may provide a better 545 understanding of what limits a population's range, while at a finer scale, N-mixture modeling offers 546 more detailed insight into how intensely used certain habitats are within a species' range". I don't think, however, that this is question of "scale". As the authors themselves state, occupancy can be used to investigate factors affecting presence-absence, while n-mixture factors affecting relative abundance. That's the difference – not one of scale. I understand what I believe is their underlying point – that occupancy works better at larger spatial scales, due to this being 1/0, while n-mixture modelling *could* be more effective at finer spatial scales due to its ability to model count data, and therefore identify differences in habitat use that occupancy cannot – but as it stands the authors are making comparisons about scale that are not backed up by their study, both modelling approaches were applied to the same spatial scale (as sampling sites were the same). I would therefore strongly recommend shifting the focus from *scale* to discussing how both methods can be

used to gain complementary insights into a population, in the form of how factors affect occurrence *and* relative abundance of a species in the study area.

Additional minor issues & comments

Across: The authors use “habitat use” and “habitat selection” interchangeably throughout, and discuss how occupancy and n-mixture modelling can be used to study habitat selection of target species. However, I don’t think this is correct – any HSM tool, as occupancy is, gives information on habitat *use*, not *selection*, as the latter implies a preference factor which we do not know is necessarily there. It’s a small distinction, but I think an important one. I would therefore suggest the authors use “habitat use” only throughout the manuscript if referring to the observed occurrence-covariate relationships.

Fig. 1 – A bit more detail on the legend. What’s the green? Forest?

111 – I think you need to caution your statements about the relative abundance and density of each species a bit better. You do not have comparable density or abundance estimate; I don’t doubt there are more bears and wolves than lynxes, but you state that beras are “far more abundant” than wolves, yet you present two metrics that are different. Again, I’m not saying that’s not necessarily the case, but I would rephrase this section to acknowledge limitation and better justify your statements.

121 – A bit unclear; what was randomly selected?

124 – 81 days in what? Per year every year? Or overall over 11 years?

129 – Covariate choice should have its own sub-section, separate from the field survey/data collection. Also, at what scale were they extracted? What was the site size? See major comment above

141 – Don’t capitalize “Occupancy”

148-149 – Unclear what this means

191 – Why is this brought up here, and why is it mentioned here even though it’s relevant to both n-mixture and occupancy?

317 – Vehicle activity; how was this done? Were all cameras on road? If not, include detection covariate? Again, see major comment above.

409: “Overall, the N-mixture model was more sensitive to factors affecting fine-scale habitat usage” What does this mean? We haven’t introduced the ideas of scale? See above – I’m not convinced you can speak about spatial scales with your results.

418: Again, please caveat that lower abundance is an assumption, and not something confirmed. Otherwise, if basing it on camera trap captures, I would suggest saying “relative abundance”.

436: “Total abundance was lower” – again, making some statements that I simply don’t think you have the data to make, even if this may be the case! I would rephrase to acknowledge this.

442: Full stop missing

460 – 462: Again, see major point above – I’m not sure it’s a question of scale; at least, this is not what you are testing. I would rephrase as suggested above.

500: “or that these species react similarly to both small and large-scale changes on the landscape.” Same point

553: “We found that N-mixture results 534 were more sensitive for all species, but increasingly so as a species’ abundance increased” See major point above – not convinced by this interpretation.

Fig. 5 – Can’t really see much here.
